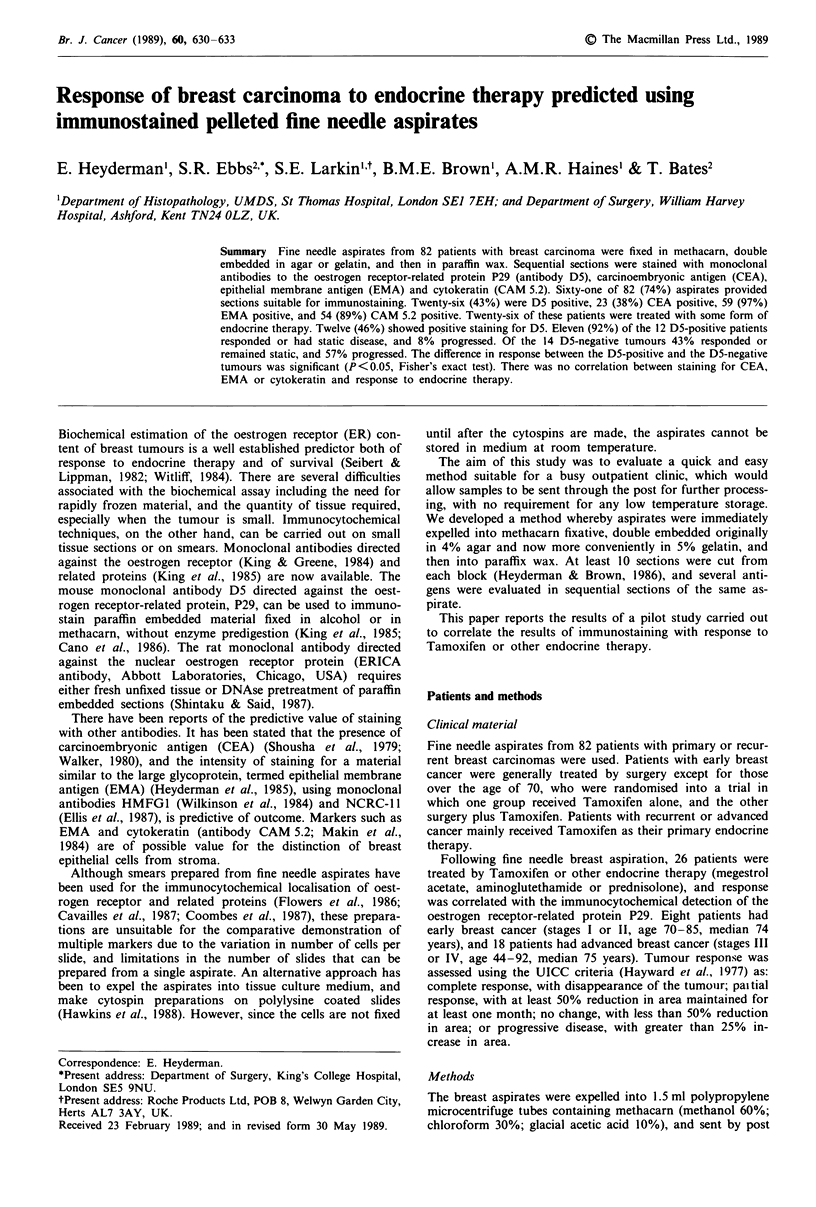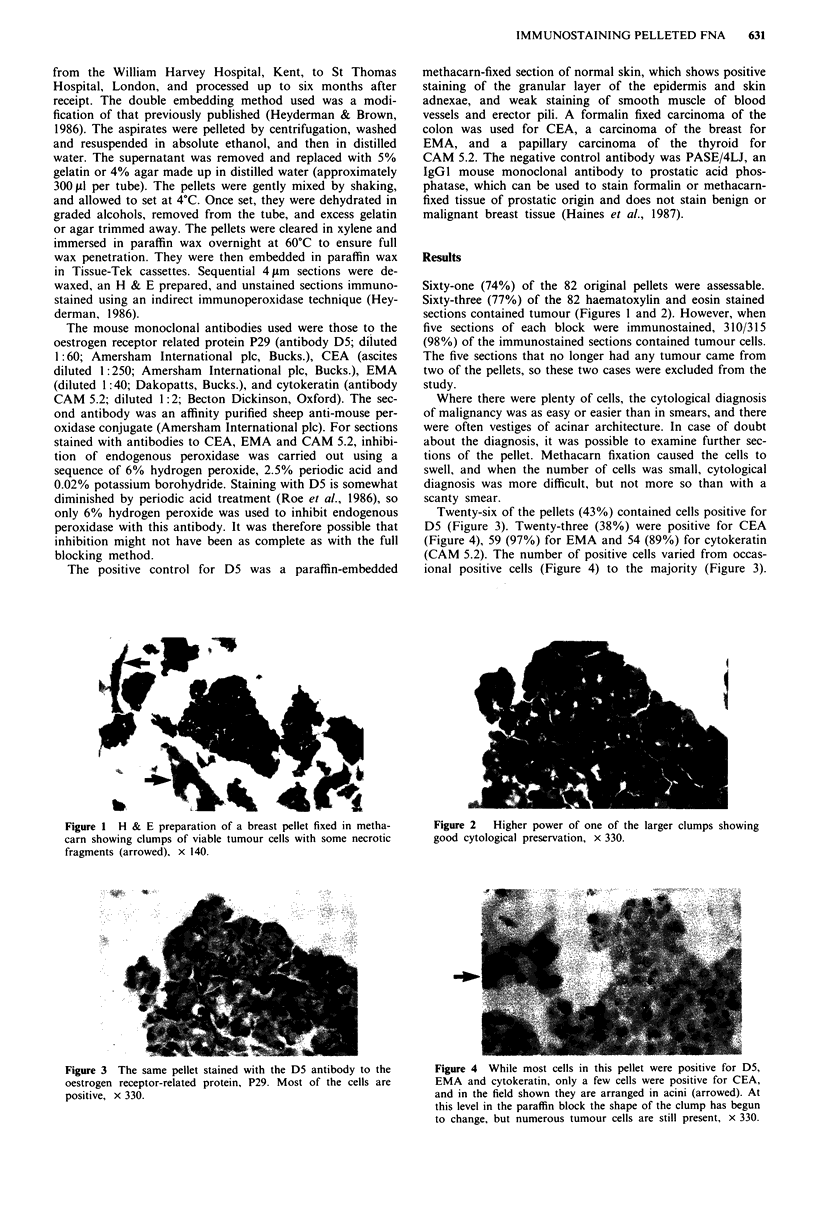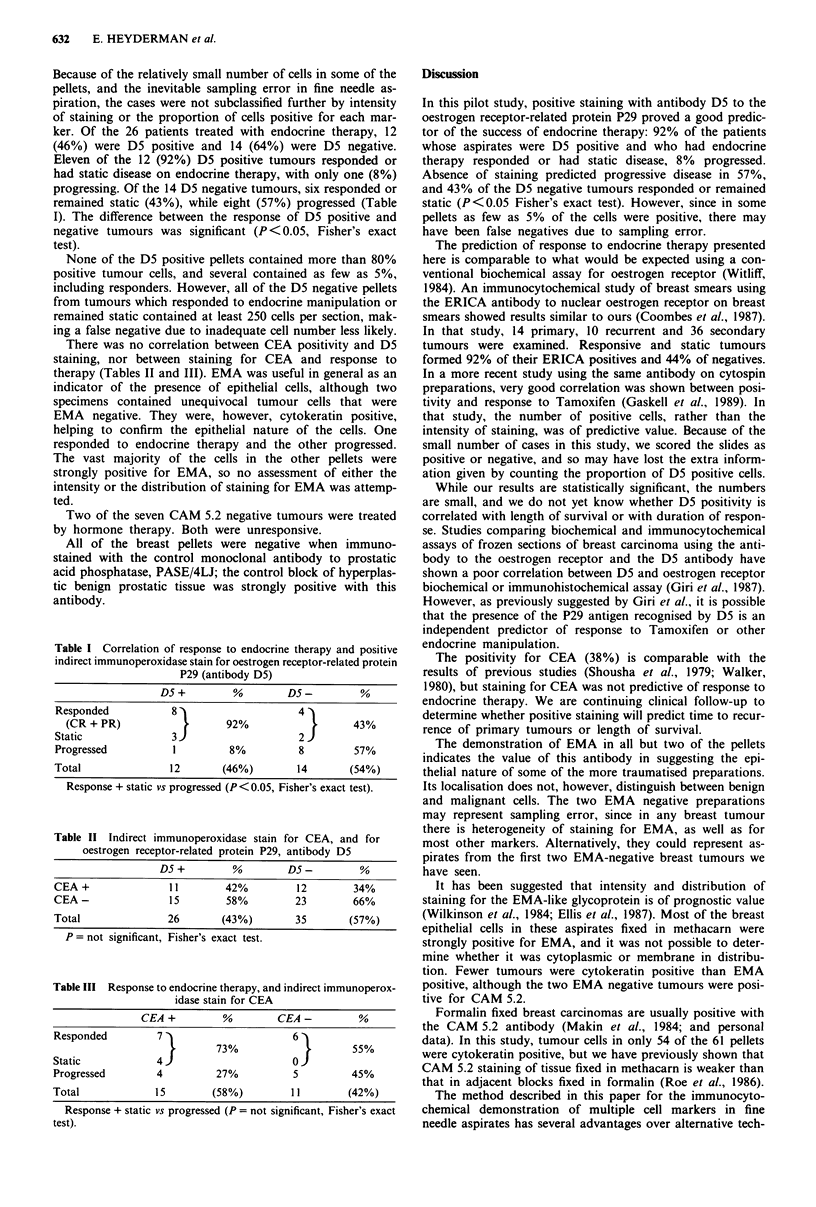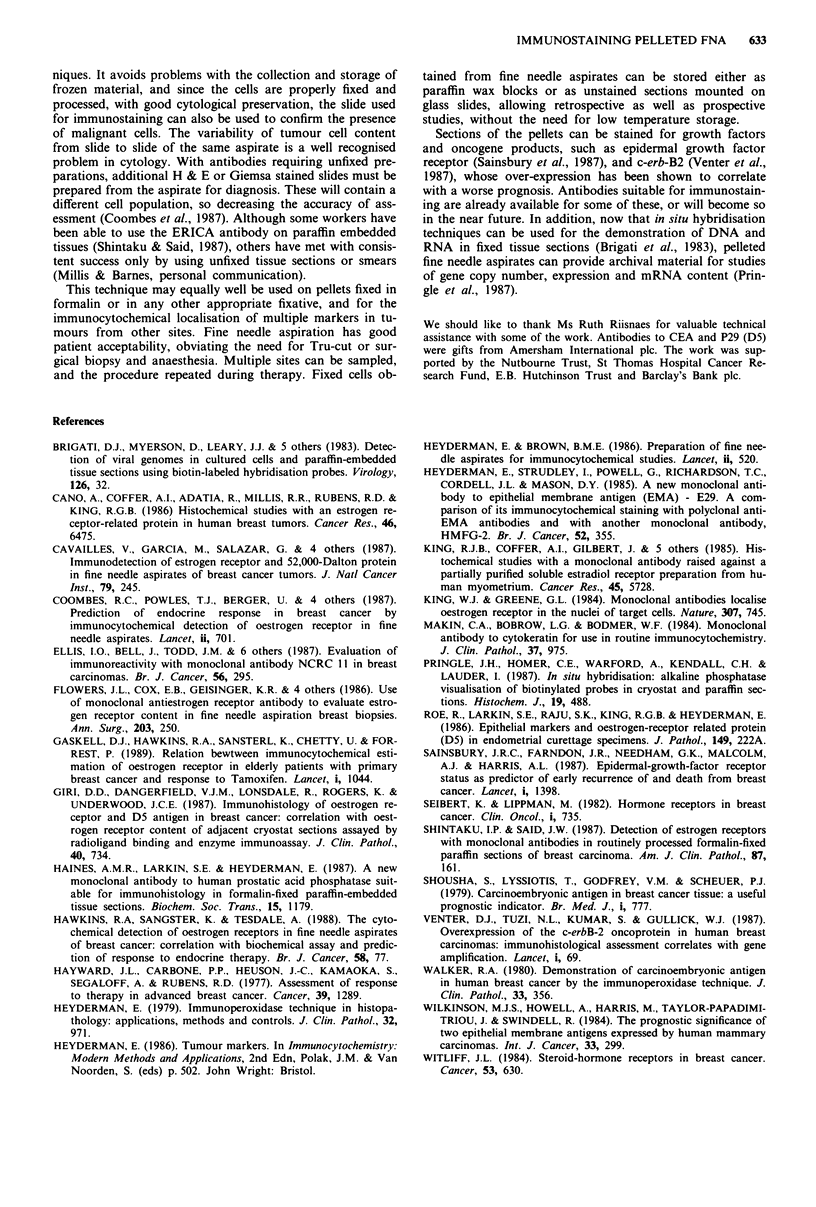# Response of breast carcinoma to endocrine therapy predicted using immunostained pelleted fine needle aspirates.

**DOI:** 10.1038/bjc.1989.328

**Published:** 1989-10

**Authors:** E. Heyderman, S. R. Ebbs, S. E. Larkin, B. M. Brown, A. M. Haines, T. Bates

**Affiliations:** Department of Histopathology, UMDS, St Thomas Hospital, London, UK.

## Abstract

**Images:**


 © The Macmillan Press Ltd., 1989

### Response of breast carcinoma to endocrine therapy predicted using immunostained pelleted fine needle aspirates

E. Heyderman[1], S.R. Ebbs[2,*], S.E. Larkin[1,†], B.M.E. Brown[1], A.M.R. Haines[1] & T. Bates[2]

[1]*Department of Histopathology, UMDS, St Thomas Hospital, London SE1 7EH; and Department of Surgery, William Harvey Hospital, Ashford, Kent TN24 0LZ, UK.*

**Summary** Fine needle aspirates from 82 patients with breast carcinoma were fixed in methacarn, double embedded in agar or gelatin, and then in paraffin wax. Sequential sections were stained with monoclonal antibodies to the oestrogen receptor-related protein P29 (antibody D5), carcinoembryonic antigen (CEA), epithelial membrane antigen (EMA) and cytokeratin (CAM 5.2). Sixty-one of 82 (74%) aspirates provided sections suitable for immunostaining. Twenty-six (43%) were D5 positive, 23 (38%) CEA positive, 59 (97%) EMA positive, and 54 (89%) CAM 5.2 positive. Twenty-six of these patients were treated with some form of endocrine therapy. Twelve (46%) showed positive staining for D5. Eleven (92%) of the 12 D5-positive patients responded or had static disease, and 8% progressed. Of the 14 D5-negative tumours 43% responded or remained static, and 57% progressed. The difference in response between the D5-positive and the D5-negative tumours was significant ($P<0.05$, Fisher's exact test). There was no correlation between staining for CEA, EMA or cytokeratin and response to endocrine therapy.

Biochemical estimation of the oestrogen receptor (ER) content of breast tumours is a well established predictor both of response to endocrine therapy and of survival (Seibert & Lippman, 1982; Witliff, 1984). There are several difficulties associated with the biochemical assay including the need for rapidly frozen material, and the quantity of tissue required, especially when the tumour is small. Immunocytochemical techniques, on the other hand, can be carried out on small tissue sections or on smears. Monoclonal antibodies directed against the oestrogen receptor (King & Greene, 1984) and related proteins (King *et al.*, 1985) are now available. The mouse monoclonal antibody D5 directed against the oestrogen receptor-related protein, P29, can be used to immunostain paraffin embedded material fixed in alcohol or in methacarn, without enzyme predigestion (King *et al.*, 1985; Cano *et al.*, 1986). The rat monoclonal antibody directed against the nuclear oestrogen receptor protein (ERICA antibody, Abbott Laboratories, Chicago, USA) requires either fresh unfixed tissue or DNAse pretreatment of paraffin embedded sections (Shintaku & Said, 1987).

There have been reports of the predictive value of staining with other antibodies. It has been stated that the presence of carcinoembryonic antigen (CEA) (Shousha *et al.*, 1979; Walker, 1980), and the intensity of staining for a material similar to the large glycoprotein, termed epithelial membrane antigen (EMA) (Heyderman *et al.*, 1985), using monoclonal antibodies HMFG1 (Wilkinson *et al.*, 1984) and NCRC-11 (Ellis *et al.*, 1987), is predictive of outcome. Markers such as EMA and cytokeratin (antibody CAM 5.2; Makin *et al.*, 1984) are of possible value for the distinction of breast epithelial cells from stroma.

Although smears prepared from fine needle aspirates have been used for the immunocytochemical localisation of oestrogen receptor and related proteins (Flowers *et al.*, 1986; Cavailles *et al.*, 1987; Coombes *et al.*, 1987), these preparations are unsuitable for the comparative demonstration of multiple markers due to the variation in number of cells per slide, and limitations in the number of slides that can be prepared from a single aspirate. An alternative approach has been to expel the aspirates into tissue culture medium, and make cytospin preparations on polylysine coated slides (Hawkins *et al.*, 1988). However, since the cells are not fixed until after the cytospins are made, the aspirates cannot be stored in medium at room temperature.

The aim of this study was to evaluate a quick and easy method suitable for a busy outpatient clinic, which would allow samples to be sent through the post for further processing, with no requirement for any low temperature storage. We developed a method whereby aspirates were immediately expelled into methacarn fixative, double embedded originally in 4% agar and now more conveniently in 5% gelatin, and then into paraffix wax. At least 10 sections were cut from each block (Heyderman & Brown, 1986), and several antigens were evaluated in sequential sections of the same aspirate.

This paper reports the results of a pilot study carried out to correlate the results of immunostaining with response to Tamoxifen or other endocrine therapy.

### Patients and methods

### *Clinical material*

Fine needle aspirates from 82 patients with primary or recurrent breast carcinomas were used. Patients with early breast cancer were generally treated by surgery except for those over the age of 70, who were randomised into a trial in which one group received Tamoxifen alone, and the other surgery plus Tamoxifen. Patients with recurrent or advanced cancer mainly received Tamoxifen as their primary endocrine therapy.

Following fine needle breast aspiration, 26 patients were treated by Tamoxifen or other endocrine therapy (megestrol acetate, aminoglutethamide or prednisolone), and response was correlated with the immunocytochemical detection of the oestrogen receptor-related protein P29. Eight patients had early breast cancer (stages I or II, age 70-85, median 74 years), and 18 patients had advanced breast cancer (stages III or IV, age 44-92, median 75 years). Tumour response was assessed using the UICC criteria (Hayward *et al.*, 1977) as: complete response, with disappearance of the tumour; paitial response, with at least 50% reduction in area maintained for at least one month; no change, with less than 50% reduction in area; or progressive disease, with greater than 25% increase in area.

### *Methods*

The breast aspirates were expelled into 1.5 ml polypropylene microcentrifuge tubes containing methacarn (methanol 60%; chloroform 30%; glacial acetic acid 10%), and sent by post

Correspondence: E. Heyderman.
*Present address: Department of Surgery, King's College Hospital, London SE5 9NU.
†Present address: Roche Products Ltd, POB 8, Welwyn Garden City, Herts AL7 3AY, UK.
Received 23 February 1989; and in revised form 30 May 1989.

from the William Harvey Hospital, Kent, to St Thomas Hospital, London, and processed up to six months after receipt. The double embedding method used was a modification of that previously published (Heyderman & Brown, 1986). The aspirates were pelleted by centrifugation, washed and resuspended in absolute ethanol, and then in distilled water. The supernatant was removed and replaced with 5% gelatin or 4% agar made up in distilled water (approximately 300 μl per tube). The pellets were gently mixed by shaking, and allowed to set at 4°C. Once set, they were dehydrated in graded alcohols, removed from the tube, and excess gelatin or agar trimmed away. The pellets were cleared in xylene and immersed in paraffin wax overnight at 60°C to ensure full wax penetration. They were then embedded in paraffin wax in Tissue-Tek cassettes. Sequential 4 μm sections were dewaxed, an H & E prepared, and unstained sections immunostained using an indirect immunoperoxidase technique (Heyderman, 1986).

The mouse monoclonal antibodies used were those to the oestrogen receptor related protein P29 (antibody D5; diluted 1:60; Amersham International plc, Bucks.), CEA (ascites diluted 1:250; Amersham International plc, Bucks.), EMA (diluted 1:40; Dakopatts, Bucks.), and cytokeratin (antibody CAM 5.2; diluted 1:2; Becton Dickinson, Oxford). The second antibody was an affinity purified sheep anti-mouse peroxidase conjugate (Amersham International plc). For sections stained with antibodies to CEA, EMA and CAM 5.2, inhibition of endogenous peroxidase was carried out using a sequence of 6% hydrogen peroxide, 2.5% periodic acid and 0.02% potassium borohydride. Staining with D5 is somewhat diminished by periodic acid treatment (Roe *et al.*, 1986), so only 6% hydrogen peroxide was used to inhibit endogenous peroxidase with this antibody. It was therefore possible that inhibition might not have been as complete as with the full blocking method.

The positive control for D5 was a paraffin-embedded methacarn-fixed section of normal skin, which shows positive staining of the granular layer of the epidermis and skin adnexae, and weak staining of smooth muscle of blood vessels and erector pili. A formalin fixed carcinoma of the colon was used for CEA, a carcinoma of the breast for EMA, and a papillary carcinoma of the thyroid for CAM 5.2. The negative control antibody was PASE/4LJ, an IgG1 mouse monoclonal antibody to prostatic acid phosphatase, which can be used to stain formalin or methacarn-fixed tissue of prostatic origin and does not stain benign or malignant breast tissue (Haines *et al.*, 1987).

### Results

Sixty-one (74%) of the 82 original pellets were assessable. Sixty-three (77%) of the 82 haematoxylin and eosin stained sections contained tumour (Figures 1 and 2). However, when five sections of each block were immunostained, 310/315 (98%) of the immunostained sections contained tumour cells. The five sections that no longer had any tumour came from two of the pellets, so these two cases were excluded from the study.

Where there were plenty of cells, the cytological diagnosis of malignancy was as easy or easier than in smears, and there were often vestiges of acinar architecture. In case of doubt about the diagnosis, it was possible to examine further sections of the pellet. Methacarn fixation caused the cells to swell, and when the number of cells was small, cytological diagnosis was more difficult, but not more so than with a scanty smear.

Twenty-six of the pellets (43%) contained cells positive for D5 (Figure 3). Twenty-three (38%) were positive for CEA (Figure 4), 59 (97%) for EMA and 54 (89%) for cytokeratin (CAM 5.2). The number of positive cells varied from occasional positive cells (Figure 4) to the majority (Figure 3).

**Figure 1** H & E preparation of a breast pellet fixed in methacarn showing clumps of viable tumour cells with some necrotic fragments (arrowed), × 140.

**Figure 2** Higher power of one of the larger clumps showing good cytological preservation, × 330.

**Figure 3** The same pellet stained with the D5 antibody to the oestrogen receptor-related protein, P29. Most of the cells are positive, × 330.

**Figure 4** While most cells in this pellet were positive for D5, EMA and cytokeratin, only a few cells were positive for CEA, and in the field shown they are arranged in acini (arrowed). At this level in the paraffin block the shape of the clump has begun to change, but numerous tumour cells are still present, × 330.

Because of the relatively small number of cells in some of the pellets, and the inevitable sampling error in fine needle aspiration, the cases were not subclassified further by intensity of staining or the proportion of cells positive for each marker. Of the 26 patients treated with endocrine therapy, 12 (46%) were D5 positive and 14 (64%) were D5 negative. Eleven of the 12 (92%) D5 positive tumours responded or had static disease on endocrine therapy, with only one (8%) progressing. Of the 14 D5 negative tumours, six responded or remained static (43%), while eight (57%) progressed (Table I). The difference between the response of D5 positive and negative tumours was significant ($P<0.05$, Fisher's exact test).

None of the D5 positive pellets contained more than 80% positive tumour cells, and several contained as few as 5%, including responders. However, all of the D5 negative pellets from tumours which responded to endocrine manipulation or remained static contained at least 250 cells per section, making a false negative due to inadequate cell number less likely.

There was no correlation between CEA positivity and D5 staining, nor between staining for CEA and response to therapy (Tables II and III). EMA was useful in general as an indicator of the presence of epithelial cells, although two specimens contained unequivocal tumour cells that were EMA negative. They were, however, cytokeratin positive, helping to confirm the epithelial nature of the cells. One responded to endocrine therapy and the other progressed. The vast majority of the cells in the other pellets were strongly positive for EMA, so no assessment of either the intensity or the distribution of staining for EMA was attempted.

Two of the seven CAM 5.2 negative tumours were treated by hormone therapy. Both were unresponsive.

All of the breast pellets were negative when immunostained with the control monoclonal antibody to prostatic acid phosphatase, PASE/4LJ; the control block of hyperplastic benign prostatic tissue was strongly positive with this antibody.

**Table I** Correlation of response to endocrine therapy and positive indirect immunoperoxidase stain for oestrogen receptor-related protein P29 (antibody D5)

| | *D5+* | % | *D5−* | % |
|---|---|---|---|---|
| Responded (CR + PR) | 8 | 92% | 4 | 43% |
| Static | 3 | | 2 | |
| Progressed | 1 | 8% | 8 | 57% |
| Total | 12 | (46%) | 14 | (54%) |

Response + static *vs* progressed ($P<0.05$, Fisher's exact test).

**Table II** Indirect immunoperoxidase stain for CEA, and for oestrogen receptor-related protein P29, antibody D5

| | *D5+* | % | *D5−* | % |
|---|---|---|---|---|
| CEA + | 11 | 42% | 12 | 34% |
| CEA − | 15 | 58% | 23 | 66% |
| Total | 26 | (43%) | 35 | (57%) |

$P$ = not significant, Fisher's exact test.

**Table III** Response to endocrine therapy, and indirect immunoperoxidase stain for CEA

| | *CEA+* | % | *CEA−* | % |
|---|---|---|---|---|
| Responded | 7 | 73% | 6 | 55% |
| Static | 4 | | 0 | |
| Progressed | 4 | 27% | 5 | 45% |
| Total | 15 | (58%) | 11 | (42%) |

Response + static *vs* progressed ($P$ = not significant, Fisher's exact test).

### Discussion

In this pilot study, positive staining with antibody D5 to the oestrogen receptor-related protein P29 proved a good predictor of the success of endocrine therapy: 92% of the patients whose aspirates were D5 positive and who had endocrine therapy responded or had static disease, 8% progressed. Absence of staining predicted progressive disease in 57%, and 43% of the D5 negative tumours responded or remained static ($P<0.05$ Fisher's exact test). However, since in some pellets as few as 5% of the cells were positive, there may have been false negatives due to sampling error.

The prediction of response to endocrine therapy presented here is comparable to what would be expected using a conventional biochemical assay for oestrogen receptor (Witliff, 1984). An immunocytochemical study of breast smears using the ERICA antibody to nuclear oestrogen receptor on breast smears showed results similar to ours (Coombes *et al.*, 1987). In that study, 14 primary, 10 recurrent and 36 secondary tumours were examined. Responsive and static tumours formed 92% of their ERICA positives and 44% of negatives. In a more recent study using the same antibody on cytospin preparations, very good correlation was shown between positivity and response to Tamoxifen (Gaskell *et al.*, 1989). In that study, the number of positive cells, rather than the intensity of staining, was of predictive value. Because of the small number of cases in this study, we scored the slides as positive or negative, and so may have lost the extra information given by counting the proportion of D5 positive cells.

While our results are statistically significant, the numbers are small, and we do not yet know whether D5 positivity is correlated with length of survival or with duration of response. Studies comparing biochemical and immunocytochemical assays of frozen sections of breast carcinoma using the antibody to the oestrogen receptor and the D5 antibody have shown a poor correlation between D5 and oestrogen receptor biochemical or immunohistochemical assay (Giri *et al.*, 1987). However, as previously suggested by Giri *et al.*, it is possible that the presence of the P29 antigen recognised by D5 is an independent predictor of response to Tamoxifen or other endocrine manipulation.

The positivity for CEA (38%) is comparable with the results of previous studies (Shousha *et al.*, 1979; Walker, 1980), but staining for CEA was not predictive of response to endocrine therapy. We are continuing clinical follow-up to determine whether positive staining will predict time to recurrence of primary tumours or length of survival.

The demonstration of EMA in all but two of the pellets indicates the value of this antibody in suggesting the epithelial nature of some of the more traumatised preparations. Its localisation does not, however, distinguish between benign and malignant cells. The two EMA negative preparations may represent sampling error, since in any breast tumour there is heterogeneity of staining for EMA, as well as for most other markers. Alternatively, they could represent aspirates from the first two EMA-negative breast tumours we have seen.

It has been suggested that intensity and distribution of staining for the EMA-like glycoprotein is of prognostic value (Wilkinson *et al.*, 1984; Ellis *et al.*, 1987). Most of the breast epithelial cells in these aspirates fixed in methacarn were strongly positive for EMA, and it was not possible to determine whether it was cytoplasmic or membrane in distribution. Fewer tumours were cytokeratin positive than EMA positive, although the two EMA negative tumours were positive for CAM 5.2.

Formalin fixed breast carcinomas are usually positive with the CAM 5.2 antibody (Makin *et al.*, 1984; and personal data). In this study, tumour cells in only 54 of the 61 pellets were cytokeratin positive, but we have previously shown that CAM 5.2 staining of tissue fixed in methacarn is weaker than that in adjacent blocks fixed in formalin (Roe *et al.*, 1986).

The method described in this paper for the immunocytochemical demonstration of multiple cell markers in fine needle aspirates has several advantages over alternative techniques. It avoids problems with the collection and storage of frozen material, and since the cells are properly fixed and processed, with good cytological preservation, the slide used for immunostaining can also be used to confirm the presence of malignant cells. The variability of tumour cell content from slide to slide of the same aspirate is a well recognised problem in cytology. With antibodies requiring unfixed preparations, additional H & E or Giemsa stained slides must be prepared from the aspirate for diagnosis. These will contain a different cell population, so decreasing the accuracy of assessment (Coombes *et al.*, 1987). Although some workers have been able to use the ERICA antibody on paraffin embedded tissues (Shintaku & Said, 1987), others have met with consistent success only by using unfixed tissue sections or smears (Millis & Barnes, personal communication).

This technique may equally well be used on pellets fixed in formalin or in any other appropriate fixative, and for the immunocytochemical localisation of multiple markers in tumours from other sites. Fine needle aspiration has good patient acceptability, obviating the need for Tru-cut or surgical biopsy and anaesthesia. Multiple sites can be sampled, and the procedure repeated during therapy. Fixed cells obtained from fine needle aspirates can be stored either as paraffin wax blocks or as unstained sections mounted on glass slides, allowing retrospective as well as prospective studies, without the need for low temperature storage.

Sections of the pellets can be stained for growth factors and oncogene products, such as epidermal growth factor receptor (Sainsbury *et al.*, 1987), and c-*erb*-B2 (Venter *et al.*, 1987), whose over-expression has been shown to correlate with a worse prognosis. Antibodies suitable for immunostaining are already available for some of these, or will become so in the near future. In addition, now that *in situ* hybridisation techniques can be used for the demonstration of DNA and RNA in fixed tissue sections (Brigati *et al.*, 1983), pelleted fine needle aspirates can provide archival material for studies of gene copy number, expression and mRNA content (Pringle *et al.*, 1987).

We should like to thank Ms Ruth Riisnaes for valuable technical assistance with some of the work. Antibodies to CEA and P29 (D5) were gifts from Amersham International plc. The work was supported by the Nutbourne Trust, St Thomas Hospital Cancer Research Fund, E.B. Hutchinson Trust and Barclay's Bank plc.

### References

BRIGATI, D.J., MYERSON, D., LEARY, J.J. & 5 others (1983). Detection of viral genomes in cultured cells and paraffin-embedded tissue sections using biotin-labeled hybridisation probes. *Virology*, **126**, 32.

CANO, A., COFFER, A.I., ADATIA, R., MILLIS, R.R., RUBENS, R.D. & KING, R.G.B. (1986) Histochemical studies with an estrogen receptor-related protein in human breast tumors. *Cancer Res.*, **46**, 6475.

CAVAILLES, V., GARCIA, M., SALAZAR, G. & 4 others (1987). Immunodetection of estrogen receptor and 52,000-Dalton protein in fine needle aspirates of breast cancer tumors. *J. Natl Cancer Inst.*, **79**, 245.

COOMBES, R.C., POWLES, T.J., BERGER, U. & 4 others (1987). Prediction of endocrine response in breast cancer by immunocytochemical detection of oestrogen receptor in fine needle aspirates. *Lancet*, **ii**, 701.

ELLIS, I.O., BELL, J., TODD, J.M. & 6 others (1987). Evaluation of immunoreactivity with monoclonal antibody NCRC 11 in breast carcinomas. *Br. J. Cancer*, **56**, 295.

FLOWERS, J.L., COX, E.B., GEISINGER, K.R. & 4 others (1986). Use of monoclonal antiestrogen receptor antibody to evaluate estrogen receptor content in fine needle aspiration breast biopsies. *Ann. Surg.*, **203**, 250.

GASKELL, D.J., HAWKINS, R.A., SANSTERL, K., CHETTY, U. & FORREST, P. (1989). Relation bewtween immunocytochemical estimation of oestrogen receptor in elderly patients with primary breast cancer and response to Tamoxifen. *Lancet*, **i**, 1044.

GIRI, D.D., DANGERFIELD, V.J.M., LONSDALE, R., ROGERS, K. & UNDERWOOD, J.C.E. (1987). Immunohistology of oestrogen receptor and D5 antigen in breast cancer: correlation with oestrogen receptor content of adjacent cryostat sections assayed by radioligand binding and enzyme immunoassay. *J. Clin. Pathol.*, **40**, 734.

HAINES, A.M.R., LARKIN, S.E. & HEYDERMAN, E. (1987). A new monoclonal antibody to human prostatic acid phosphatase suitable for immunohistology in formalin-fixed paraffin-embedded tissue sections. *Biochem. Soc. Trans.*, **15**, 1179.

HAWKINS, R.A, SANGSTER, K. & TESDALE, A. (1988). The cytochemical detection of oestrogen receptors in fine needle aspirates of breast cancer: correlation with biochemical assay and prediction of response to endocrine therapy. *Br. J. Cancer*, **58**, 77.

HAYWARD, J.L., CARBONE, P.P., HEUSON, J.-C., KAMAOKA, S., SEGALOFF, A. & RUBENS, R.D. (1977). Assessment of response to therapy in advanced breast cancer. *Cancer*, **39**, 1289.

HEYDERMAN, E. (1979). Immunoperoxidase technique in histopathology: applications, methods and controls. *J. Clin. Pathol.*, **32**, 971.

HEYDERMAN, E. (1986). Tumour markers. In *Immunocytochemistry: Modern Methods and Applications*, 2nd Edn, Polak, J.M. & Van Noorden, S. (eds) p. 502. John Wright: Bristol.

HEYDERMAN, E. & BROWN, B.M.E. (1986). Preparation of fine needle aspirates for immunocytochemical studies. *Lancet*, **ii**, 520.

HEYDERMAN, E., STRUDLEY, I., POWELL, G., RICHARDSON, T.C., CORDELL, J.L. & MASON, D.Y. (1985). A new monoclonal antibody to epithelial membrane antigen (EMA) - E29. A comparison of its immunocytochemical staining with polyclonal anti-EMA antibodies and with another monoclonal antibody, HMFG-2. *Br. J. Cancer*, **52**, 355.

KING, R.J.B., COFFER, A.I., GILBERT, J. & 5 others (1985). Histochemical studies with a monoclonal antibody raised against a partially purified soluble estradiol receptor preparation from human myometrium. *Cancer Res.*, **45**, 5728.

KING, W.J. & GREENE, G.L. (1984). Monoclonal antibodies localise oestrogen receptor in the nuclei of target cells. *Nature*, **307**, 745.

MAKIN, C.A., BOBROW, L.G. & BODMER, W.F. (1984). Monoclonal antibody to cytokeratin for use in routine immunocytochemistry. *J. Clin. Pathol.*, **37**, 975.

PRINGLE, J.H., HOMER, C.E., WARFORD, A., KENDALL, C.H. & LAUDER, I. (1987). *In situ* hybridisation: alkaline phosphatase visualisation of biotinylated probes in cryostat and paraffin sections. *Histochem. J.*, **19**, 488.

ROE, R., LARKIN, S.E., RAJU, S.K., KING, R.G.B. & HEYDERMAN, E. (1986). Epithelial markers and oestrogen-receptor related protein (D5) in endometrial curettage specimens. *J. Pathol.*, **149**, 222A.

SAINSBURY, J.R.C., FARNDON, J.R., NEEDHAM, G.K., MALCOLM, A.J. & HARRIS, A.L. (1987). Epidermal-growth-factor receptor status as predictor of early recurrence of and death from breast cancer. *Lancet*, **i**, 1398.

SEIBERT, K. & LIPPMAN, M. (1982). Hormone receptors in breast cancer. *Clin. Oncol.*, **i**, 735.

SHINTAKU, I.P. & SAID, J.W. (1987). Detection of estrogen receptors with monoclonal antibodies in routinely processed formalin-fixed paraffin sections of breast carcinoma. *Am. J. Clin. Pathol.*, **87**, 161.

SHOUSHA, S., LYSSIOTIS, T., GODFREY, V.M. & SCHEUER, P.J. (1979). Carcinoembryonic antigen in breast cancer tissue: a useful prognostic indicator. *Br. Med. J.*, **i**, 777.

VENTER, D.J., TUZI, N.L., KUMAR, S. & GULLICK, W.J. (1987). Overexpression of the c-*erb*B-2 oncoprotein in human breast carcinomas: immunohistological assessment correlates with gene amplification. *Lancet*, **i**, 69.

WALKER, R.A. (1980). Demonstration of carcinoembryonic antigen in human breast cancer by the immunoperoxidase technique. *J. Clin. Pathol.*, **33**, 356.

WILKINSON, M.J.S., HOWELL, A., HARRIS, M., TAYLOR-PAPADIMITRIOU, J. & SWINDELL, R. (1984). The prognostic significance of two epithelial membrane antigens expressed by human mammary carcinomas. *Int. J. Cancer*, **33**, 299.

WITLIFF, J.L. (1984). Steroid-hormone receptors in breast cancer. *Cancer*, **53**, 630.